# Machine Learning-Based Retention Time Prediction of Trimethylsilyl Derivatives of Metabolites

**DOI:** 10.3390/biomedicines10040879

**Published:** 2022-04-11

**Authors:** Sara M. de Cripan, Adrià Cereto-Massagué, Pol Herrero, Andrei Barcaru, Núria Canela, Xavier Domingo-Almenara

**Affiliations:** 1 Computational Metabolomics for Systems Biology Lab, Omics Sciences Unit, Eurecat—Technology Centre of Catalonia, 08005 Barcelona, Catalonia, Spain; sara.martinez@eurecat.org; 2 Centre for Omics Sciences (COS), Eurecat—Technology Centre of Catalonia & Rovira i Virgili University Joint Unit, Unique Scientific and Technical Infrastructures (ICTS), 43204 Reus, Catalonia, Spain; adria.cereto@ce.eurecat.org (A.C.-M.); pol.herrero@eurecat.org (P.H.); nuria.canela@eurecat.org (N.C.); 3 Department of Electrical, Electronic and Control Engineering (DEEEA), Universitat Rovira i Virgili, 43007 Tarragona, Catalonia, Spain; 4 Independent Researcher, 1012 WX Amsterdam, The Netherlands; andrei_barcaru@yahoo.com

**Keywords:** retention time, retention index, GC-MS, metabolomics, machine-learning

## Abstract

In gas chromatography–mass spectrometry-based untargeted metabolomics, metabolites are identified by comparing mass spectra and chromatographic retention time with reference databases or standard materials. In that sense, machine learning has been used to predict the retention time of metabolites lacking reference data. However, the retention time prediction of trimethylsilyl derivatives of metabolites, typically analyzed in untargeted metabolomics using gas chromatography, has been poorly explored. Here, we provide a rationalized framework for machine learning-based retention time prediction of trimethylsilyl derivatives of metabolites in gas chromatography. We compared different machine learning paradigms, in addition to exploring the influence of the computational molecular structure representation to train the prediction models: fingerprint class and fingerprint calculation software. Our study challenged predicted retention time when using chemical ionization and electron impact ionization sources in simulated and real cases, demonstrating a good correct identity ranking capability by machine learning, despite observing a limited false identity filtering power in cases where a spectrum or a monoisotopic mass match to multiple candidates. Specifically, machine learning prediction yielded median absolute and relative retention index (relative retention time) errors of 37.1 retention index units and 2%, respectively. In addition, fingerprint class and fingerprint calculation software, as well as the molecular structural similarity between the training and test or real case sets, showed to be critical modulators of the prediction performance. Finally, we leveraged the structural similarity between the training and test or real case set to determine the probability that the prediction error is below a specific threshold. Overall, our study demonstrates that predicted retention time can provide insights into the true structure of unknown metabolites by ranking from the most to the least plausible molecular identity, and sets the guidelines to assess the confidence in metabolite identification using predicted retention time data.

## 1. Introduction

Beyond the historical application of metabolomics for biomarker discovery, metabolomics is now being recognized for its potential for mechanism elucidation [1] or the discovery of bioactive metabolites [2]. Although limited in metabolite coverage compared to liquid chromatography–mass spectrometry (LC–MS), gas chromatography coupled to mass spectrometry (GC–MS) is a widely used analytical platform in untargeted metabolomics for volatile and semi-volatile metabolite measurement thanks to the robustness of the capillary columns used in GC, its relatively simple maintenance, the highly reproducible electron impact (EI) source or the “softness” of the chemical ionization (CI) source [3].

Currently, molecular identification is one of the most important limitations in metabolomics [4,5]. The metabolomics standards initiative (MSI) guidelines state that a specific metabolite in an experimental sample is identified when two orthogonal properties (e.g., retention time (RT) and mass spectra (MS)) match to those of an authentic pure standard analyzed under identical analytical conditions [6]. Thus, RT is routinely used in both liquid or gas chromatography coupled to mass spectrometry for metabolite identification. However, not all the molecules have commercially available pure standards and alternatives to generate RT data for commercially unavailable metabolites are needed.

The molecule’s RT depends on multiple variables, including the column type and length, phase ratios or temperature program, among others [7]. However, the robustness of the capillary columns has facilitated the adoption of a retention index (RI) calibration method. RI data are available in libraries and databases and encode the metabolites’ relative retention time differences to a set of reference standards such as n-alkanes or fatty acid methyl esters (FAMEs). These standards are co-injected with the experimental samples, but methods that bypass the need for reference standard co-injection –using instead naturally occurring metabolites for calibration– also exist [8]. Thus, RI data enable confident metabolite annotation by comparison of observed with expected RI.

Despite the utility of RI, few RI databases exist, being the NIST the most comprehensive existing library to date. To overcome this limitation, machine learning (ML) strategies have been designed to generate RI for any given molecule [9,10,11]. These strategies have been focused on RI prediction of volatile molecules, of relevance in many fields including toxicology, pathology, biomarker discovery or plant extracts and metabolome research [10,12,13]. This can be mainly attributed to the availability of large RI data in the NIST library since large datasets -from which an ML model can learn- are necessary to train and build accurate ML models [14,15]. However, in GC–MS-based metabolomics, compounds in samples are typically derivatized. Derivatization is required to make metabolites more volatile while protecting them from thermal degradation from high GC–MS temperatures [16]. The most widely adopted derivatization protocol in metabolomics uses trimethylsilylation [17,18] which removes acidic protons from hydroxyl-, carboxyl-, amino- or thiol- groups [19]. Although the NIST database covers the RI for a large number of molecules, only a small fraction corresponds to trimethylsilyl (TMS) derivatives of metabolites. Overall, RI prediction of TMS derivatives of metabolites has been poorly studied despite its potential application to routine metabolomics experiments.

To predict RI using ML, two overarching aspects of the prediction model have to be considered, being the first the type of ML approach (e.g., support vector machines (SVM), random forest (RF), deep learning (DL), to name the most popular ones), and the second, the model input (e.g., molecular descriptors, fingerprints, or a different type of representation). Molecular descriptors are a popular model input [20]. However, they provide a large number of data, sometimes redundant or highly correlated, which hampers the accurate learning by ML models. Encoding notation for molecular structures such as fingerprints (FPs)- generated using specialized software—have shown their power to predict RI values, overcoming the limitation of choosing the suitable molecular descriptors [21,22,23]. However, there are multiple free and commercial software for FP calculation, which adds another aspect to be considered.

Here we introduce an optimized ML model for RI prediction (relative RT) of TMS metabolite derivatives, trained using the Golm Metabolome Database (GMD), a specialized metabolomics library covering up to 1410 TMS metabolite derivatives. Our study compared different popular machine learning techniques as well as types of FPs and FP calculation software. To show the relevance of RI prediction in clinical metabolomics, we applied our RI prediction model for metabolite identification in a comparative analysis of plasma samples from patients with ulcerative colitis (UC) and healthy controls.

## 2. Methods

### 2.1. Fingerprint Calculation and Dataset Generation

There exists several types of molecular FP classes, reviewed elsewhere [24], depending on the method employed to transform the molecular structure into a bit string. We generated different classes of FP with different software types (see Table S1 and Supplementary Materials) using the molecular structures with SMILES representation contained in the GMD using derivatized metabolites with TMS. Out of the total 1410 compounds contained in the database we generated FPs for 1159 compounds. We discarded those compounds with incomplete information in the database (e.g., lacking SMILES) or when it was not possible to generate FP representation with some of the software types employed.

### 2.2. Machine Learning Model Training and Test

All the analyses were performed in the R environment (version 4.0) using packages freely available in the CRAN repository. Deep Neural Network (DNN) as well as Convolutional Neural Network (CNN) algorithms were deployed with R implementation of Keras (version 2.3), e1071 (version 1.7) was used to implement SVM algorithm with linear (SVM-Lin) and polynomial (SVM-Poly) kernel. Finally, a RF model was assembled with randomForest (version 4.6). Model parameters are described in Supplementary Materials.

**Figure 1:**
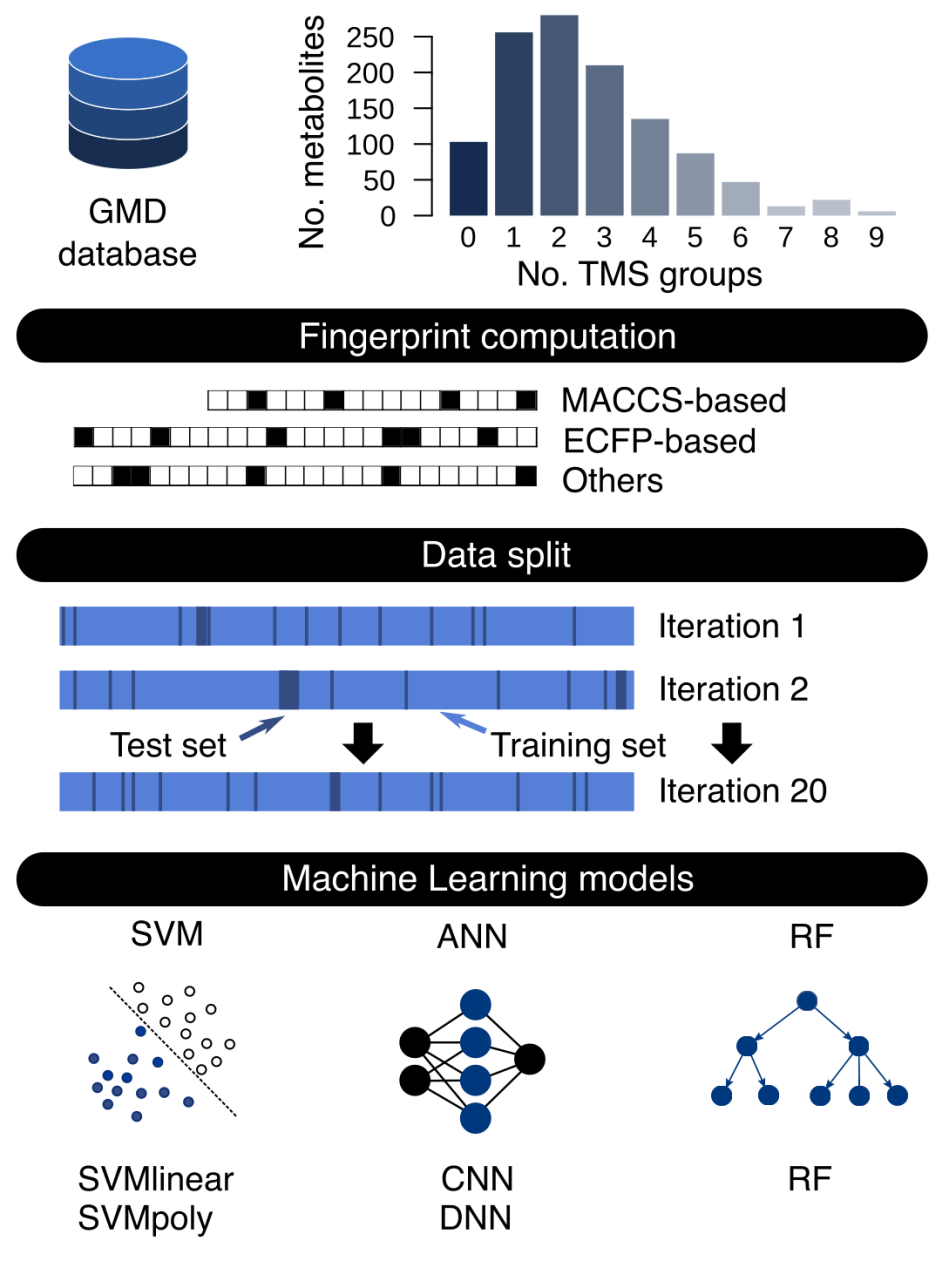
Workflow for RI prediction. The histogram shows the number of TMS groups of metabolites in GMD. Multiple fingerprint classes were generated from metabolites in GMD and metabolites were randomly split in training and test sets generating 20 different sets to train and test the ML models.

Each model was iteratively evaluated with 20 random splits of the data preserved across models, randomized as a training set with 75% of the metabolites employed to train each of the models and a test set with 25% of the compounds intended to validate the models (Figure 1). This ensured that almost every considered compound was evaluated in both training and test sets and allowed us to evaluate if similarity levels across compounds in different datasets affect the prediction accuracy. ML models benefit from certain transformations, including 10 factor scaling [25]. RI values were multiplied by 10 since this increased prediction accuracy by the models. The R scripts to reproduce the results are available at https://github.com/smdecripan/RIpred, accessed on 1 March 2022.

### 2.3. GC-MS Plasma Analysis

Details on metabolite extraction and GC-ToF-MS analysis can be found in the Supplementary Materials. GC–MS raw data (mzXML files) has been deposited in the Metabolights repository with accession number MTBLS2841. After GC–MS analysis, raw data were converted to mzXML file using Proteowizard [26] and processed by eRah (V1.1.2). eRah output consisted of a set of experimental deconvolved spectra and RI, that was matched to metabolite reference spectra and RI data from GMD. Empirical RI values were determined by using naturally occurring metabolites in samples (identified using standard materials) as internal RI reference as described elsewhere [8], to avoid the need for reference standard co-injection and to prevent cluttering chromatograms with unnecessary peaks that can otherwise mask potential metabolite peaks [8]. We manually identified a total of 62 metabolites, covering the entire RT range (min 5 to 20), using reference standards materials when available or by RI/MS comparison with reference data in GMD (<1% RI error and >80% of spectral similarity). This manual inspection consisted in discarding those cases where spectral and RI similarity among candidates was too ambiguous to assign the correct identity without standard addition or isotopically labeled standard co-injection techniques [4]. Metabolites showing high spectral similarity to reference data but with RI errors above 1% were confirmed using pure standard materials.

## 3. Results

### 3.1. TMS Derivatives of Metabolites RI Prediction via Machine Learning Models

**Figure 2:**
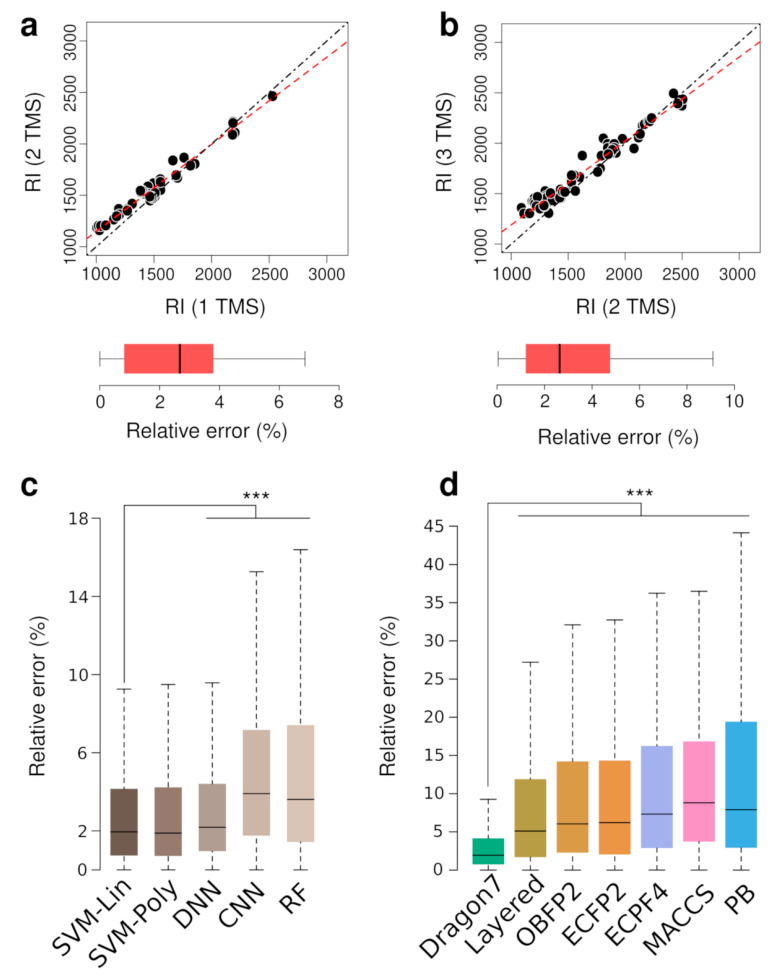
(**a**) RI of derivatized metabolites with 1TMS vs. 2TMS, (**b**) RI of derivatized metabolites with 2TMS vs. 3TMS, and box plots showing the corresponding relative prediction errors as determined by linear regression. Dashed black lines are the identity functions and the dashed red lines are the regression lines. (**c**) Prediction error for each ML model and (**d**) FP class. *p*-value < 0.001 from a paired Wilcoxon rank tests (n = 5800) is shown as ***. Outliers are not shown (all panels).

It has been shown that similar molecular structures have a similar RT and that ML-based prediction has difficulties trying to predict these differences among similar molecules [27,28]. In GC–MS-based metabolomics, the same metabolite can be observed multiple times with different TMS derivative groups (Figure 1). These molecules will have a similar RI as they share a common molecular substructure. We first studied RI differences among the same metabolites with different TMS groups to explore how their structural similarity could affect the ML’s ability to accurately predict the RI for those metabolites. Figure 2a,b shows the RI relation between the same metabolite with 1 and 2 TMS groups and 2 and 3 TMS groups. Other pairwise comparisons (e.g., 0TMS vs. 1TMS, or 1, 2 or 3TMS with 4TMS) were not performed due to the lack of data in GMD. Metabolites with 2 TMS groups showed a 6.05% RI increase compared to their 1 TMS counterparts, and metabolites with 3 TMS groups showed a 5.35% RI increase compared to their 2 TMS counterparts (Figure 2c,d). As shown in Figure 2a,b, there is a linear relation among the RI of molecules with 1 and 2 TMS groups and 2 and 3 TMS groups. By using a simple linear regression we can determine the RI of metabolites with 2 TMS or 3 TMS knowing the RI of their counterpart with one TMS group less, and vice versa. This simple prediction yielded median errors of 2.79% and 2.64% for 1 to 2 TMS and 2 to 3 TMS, respectively.

Due to machine learning models flexibility several models and strategies have been proposed to predict RI values. The first models that gained popularity included artificial neural networks (ANNs), multiple linear regression (MLR), SVM, or RF [21,29,30,31]. The development of databases containing RI data and the increase of computational resources allowed the implementation of more complex models such as CNN or DL [10,12,32]. We studied the performance of different types of machine learning algorithms with different configurations, yielding a total of five ML models: SVM algorithm with linear (SVM-Lin) and polynomial (SVM-Poly) kernel, DNN, CNN and RF (Figure 1) (see Section 2 and Supplementary Materials for details on models construction and parameters).

We used molecular FP–a computational molecular representation–as ML model input. There is a wide range of molecular FPs and FP calculation software. Therefore, we aimed at exploring whether the FP class or software has an influence on prediction accuracy. We used four different FP calculation software, including the commercial Dragon 7.0 (Kode Chemoinformatics, Pisa, Italy) or open-source tools like RDKit [33], OpenBabel [34] and ChemFP [35], to calculate seven types of popular FP configurations (combining FP class and software). These included 2- and 4-diameter Extended-Connectivity Fingerprints (ECFP) and Layered FP generated with RDKit; ECFP generated with Dragon; FP2-class FP generated with OpenBabel; and MACCS- and PubChem-class (PB) generated with ChemFP and OpenBabel as back-end (see Table S1 and Supplementary Materials for more details on FP class description). We determined the best combinations of FP and ML models after different rounds of hyperparameter optimization and testing (prediction errors are available in Table S2). Due to limited data, we iteratively evaluated each ML model with 20 random splits of the data in training and test sets (Figure 1, see Section 2 for details).

Dragon’s FP showed the best performance independently of the ML method used. Therefore, we compared the different ML models’ performance using Dragon’s FP. Models’ performance results, including Mean Average Error (MAE), Mean Average Percentage Error (MAPE), Median Average Error (MdAE) and Median Average Percentage Error (MdAPE), are shown in Table S2. As observed in Figure 2c, both SVM with linear and polynomial kernels (SVM-Lin and SVM-Poly) as well as DNN models using Dragon’s FP achieved similar prediction accuracy. The difference in performance between both SVM models was not statistically significant, but the SVM with linear kernel implementation is more interpretable than the polynomial kernel-based model. SVM-Lin MdAE was 5 RI units lower than DNN model (Table S2) and SVM-Lin median relative errors were statistically significant different from the median RI errors of DNN (paired Wilcoxon test, *p*-value < 0.001, n = 5800). A SVM linear model is favored over DNN because simpler models are prone to less overfitting. In that sense, we chose SVM with a linear kernel (SVM-Lin) as the prediction model for the rest of this study, and to assess the influence of different FP classes as input data to predict RI values (Figure 2d). Figure 2d shows that the SVM-Lin model prediction accuracy is affected by the FP class used to represent molecular structures. Dragon’s FP prediction results were statistically significant more accurate than the rest of FP (paired Wilcoxon rank test, *p*-value < 0.001, n = 5800). Of note, hyperparameter optimization for each FP did not yield significant accuracy improvements, and the parameters optimized for Dragon’s FP data gave the best results over all FP classes.

### 3.2. Training Set Structural Similarity Influence on Prediction Performance

**Figure 3:**
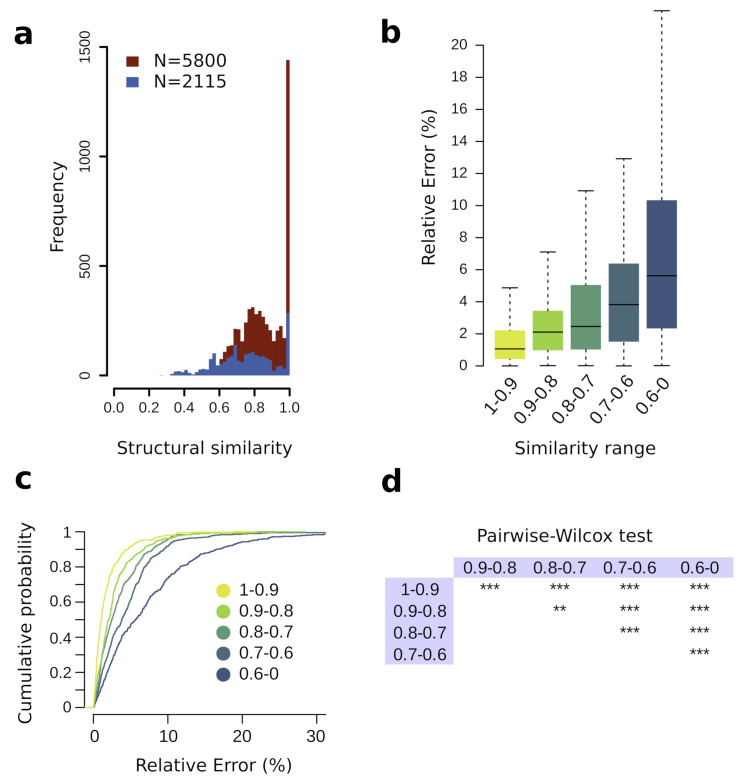
Structural similarity influence. (**a**) Distribution of Tanimoto similarity of the full training set (red) and randomly selected metabolites (blue). (**b**) Prediction error across similarity ranges. (**c**) Cumulative probability functions (CPF) for the structural similarity ranges. (**d**) Statistical significance for pairwise comparisons in (**b**): Wilcoxon test, *** for *p*-value < 0.001; ** for *p*-value < 0.01.

We aimed at assessing how metabolites included in the training set modulate the prediction performance of the SVM-Lin model. As mentioned before, it has been shown that similar molecular structures have a similar RT [27,28]. Specifically, it has been observed that ML-based RT prediction performance in LC-MS depends on the similarity level of molecules in the training set to those in the test set [15]. We explored whether this phenomenon is echoed in TMS derivatives of metabolites analyzed using GC-MS. To that end, we focused on the prediction error for those metabolites in the test set that had at least one metabolite in the training set above a specific degree of similarity. We used the Tanimoto similarity coefficient to measure the similarity among molecules. The Tanimoto similarity coefficient measures how similar the two-dimensional structures of two molecules are and it ranges from 0 (no similarity) to 1 (identical molecules). The distribution of Tanimoto score (Figure 3a) showed a peak of identical metabolites (Tanimoto score of 1) corresponding to stereoisomers present in GMD. Interestingly, there is a limited number of metabolites with Tanimoto score under 0.6. The prediction error for metabolites in the test set was divided into five groups, each group considering those metabolites in the test set that had at least one similar metabolite in the training set within a specific similarity range (1 to ⩾0.9, <0.9 to ⩾0.8, <0.8 to ⩾0.7, <0.7 to ⩾0.6, <0.6 to 0). To allow for statistically unbiased comparison among groups, we randomly selected the same number of metabolites for all groups (N = 423, corresponding to the size of the smallest group, <0.6 to 0). Figure 3b, shows the prediction error (relative error) according to the similarity scores. It can be observed that prediction accuracy increases in accordance with the similarity of the predicted metabolites (test set) to the metabolites in the training set. We performed a pairwise Mann–Whitney–Wilcoxon test and all pairwise comparisons showed statistically significant differences (*p*-value < 0.01, Figure 3c), demonstrating that metabolite similarity among training and test sets influences the prediction accuracy.

Given that structural similarity has a strong influence on the prediction performance, we hypothesized that we could estimate the prediction error for a given metabolite. This means that if we aim at predicting the RI for a specific metabolite, we can first measure the structural similarity of this metabolite with metabolites in the training set, and, according to this similarity, determine the likelihood of the prediction error for a given metabolite being lower than a specific threshold [36]. To that end, we determined the cumulative probability function (CPF) from the distribution of the observed prediction error for each structural similarity range, as determined before (Figure 3b). The CPF describes the probability that a variable has a value lower than a specific threshold, given an empirical distribution (observed data). Figure 3d shows the prediction error CPF according to the structural similarity ranges previously described. For instance, the probability of predicting an RI with a prediction error lower than 1% for a metabolite with a structural similarity value compressed between 1 and 0.9 is 48%. This probability is reduced to 25.8% if the similarity is between 0.9 and 0.8, to 24.11% for a similarity between 0.8 and 0.7, to 16.3% for a similarity between 0.7 and 0.6, and 0.11% when similarity is lower than 0.6.

### 3.3. Ranking and Filtering Capability of the ML Model

To show the application of predicted RI in metabolomics, we evaluated the performance of the proposed RI prediction model for the two widely used MS ionization methods in GC–MS, CI and EI, using GMD data as a benchmark. Multiple putative metabolite identities can match to an observed protonated/deprotonated ion in CI via accurate mass search or to an observed spectrum obtained by EI via spectral comparison. We aimed to study how predicted RI can help at filtering and ranking putative candidates in a typical situation in metabolomics where we have multiple putative candidates that match to an observed unknown ion or spectrum.

We first generated different training/test sets to avoid the bias induced by a particular training/test set configuration as previously described. We initialized 20 test sets with a specific number of randomly selected metabolites (seed metabolites). These test sets were then expanded by including those metabolites in GMD with the same monoisotopic mass as the initial seed metabolites for the CI case (below a 10 ppm error) or with similar MS spectra to the seed metabolites for the EI case (80% similarity, see Supplementary Materials for details). We will refer to metabolites with the same monoisotopic mass or with similar spectra to the initial seed metabolites as potential interfering metabolites (PIMs). The training sets were composed of the remaining metabolites (those not included in each test set). To ensure that the resulting training sets covered at least 80% of the total number of metabolites, we set the specific number of randomly selected metabolites (seed metabolites) for the test sets to 70 and 20 metabolites for EI and CI cases, respectively. This yielded 20 training sets for each case of different sizes covering from 83 to 87.7% of the total number of metabolites in the CI case and 87.7 to 96.2% in the EI case. After training, we divided all metabolites in the test set into groups composed of each seed metabolite and its corresponding PIMs. For each group, we determined the predicted-reference RI errors for all metabolites (seed and PIMs) using the reference RI value of the seed metabolite in GMD. This resulted in a ranked list of candidates according to their predicted-reference RI error, allowing us to simulate the case where a predicted RI error is used to rank all potential candidates, replacing the experimental data in real cases with reference RI data in GMD for simulation purposes. In that sense, we evaluated the SVM-Lin model performance at ranking putative identities based on their predicted-reference RI error with a special focus on the capability to rank the correct identity among the top 3 first candidates with the lowest error.

**Figure 4:**
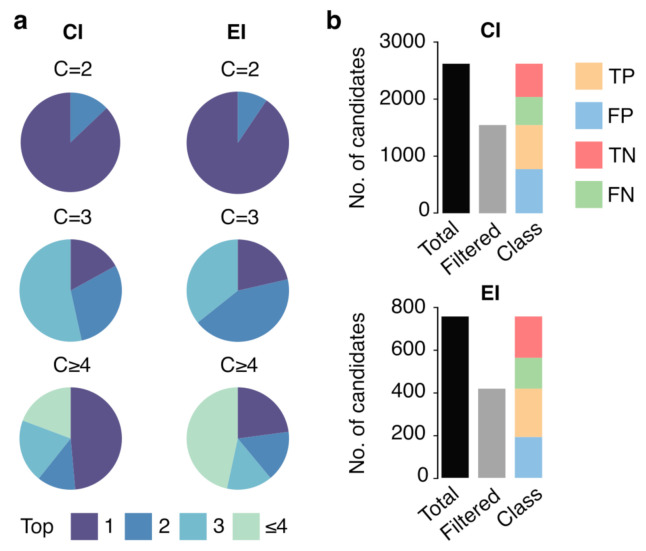
Metabolite identity candidates ranking and filtering using predicted RI in EI and CI. (**a**) Percentage of correctly identified metabolites with 2 (C = 2), 3 (C = 3), or 4 or more (C ≥ 4) putative candidates ranked according to predicted-reference RI error. (**b**) Total number of metabolites (black), number of filtered metabolites using a 3% RI threshold (gray) and candidate classification as True Positives (TP), True Negatives (TN), False Positives (FP) and False Negatives (FN) using the same threshold.

Ranking results are shown in Figure 4a. The pie charts show the percentage of cases in which the correct identity was ranked as the first, second or third candidate, based on predicted-reference RI error, for cases where there were 2 (C = 2), 3 (C = 3) or 4 or more (C ≥ 4) putative candidates in total. Results for the CI case showed that the SVM-Lin model had a good capability at ranking the true metabolite identity as the first candidate in up to 86.7% of the cases when there were two possible matches, and ranked the true metabolite identity as the first and second candidate in up to 53.5% and 29.6% of the cases, respectively, when there were three candidates. In cases where there were 4 or more candidates, the model ranked the correct identity within the top 3 candidates in 51.4% of the cases. For the EI case, ranking results showed a similar performance as the CI case, with 90.6% of the metabolites ranked as the first candidate when there were two possible matches, up to 35.7% cases as the first candidate and 42.9% as the second candidate when there were three putative candidates, and 77.1% of the candidates correctly identified within the top 3 candidates in cases where there were 4 or more candidates.

Next, we evaluated the SVM-Lin model performance at filtering the true identity from all PIMs i.e., retaining as many true positive identities as possible while filtering out as many false positive identities as possible. We aimed to filter PIMs or false positives candidates by removing all matches with a predicted-experimental RI error above a specific filtering threshold. To determine the best threshold, we used a receiver operating characteristic (ROC) curve that depicts the performance of a range of filtering thresholds at discriminating true positive, false negative, true positive or false positive identities. We calculated ROC curves by discretizing the range of the filtering thresholds from 0 to 100% in intervals of 1%. Subsequently, we determined the sensitivity or true positive rate (TPR) and the specificity or false positive rate (FPR) (see Supplementary Materials for details). ROC curves for CI and EI cases, available in Figure S1, showed area under the curve (AUC) values of 0.53 and 0.56 respectively. Based on the ROC curves, we determined an optimal filtering threshold (RI error of 3%) that allowed reducing the total number of putative candidates while minimizing the loss of true identities. This 3% threshold enabled assigning 61% of the metabolites among the three first candidates with an FPR of 43% and 50% for CI and EI cases, respectively. Figure 4b shows the distribution of true identity and the putative candidates: in the CI case, 29.5% of the candidates were classified as true positives, 22.3% as true negatives, 29.5% as false positives and 18.7% as false negatives; in EI case the classification was 30.0% as true positives, 25.6% as true negatives, 25.3% as false positives and 19.1% false negatives.

### 3.4. Application of the Prediction Model for Metabolite Identification in Plasma from Patients with Ulcerative Colitis

**Figure 5:**
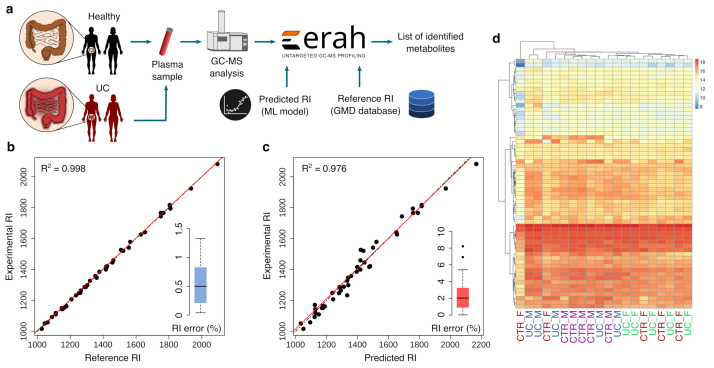
Application of RT/RI prediction in plasma samples from patients with ulcerative colitis (UC). (**a**) Workflow graphical representation. (**b**) Experimental vs. reference RI and (**c**) experimental vs. predicted RI of the identified metabolites. (**d**) Heat-map and sample hierarchical clustering of identified metabolites in plasma samples (M for male, F for female, CTR for control and UC for ulcerative colitis).

To demonstrate the utility of our prediction model in clinical applications, we used untargeted metabolomics to analyze a set of 25 plasma samples, consisting of 10 plasma samples from patients with UC and 10 samples from age-, weight-, and sex-matched healthy controls (a total of 10 males and 10 females), in addition to 5 technical replicates of a pooled sample used as quality controls (Figure 5a). Plasma samples were analyzed using GC-ToF-MS (see Section 2 and Supplementary Materials for details), and raw data were processed using eRah [37]. eRah generated a list of deconvolved EI spectra that were matched against GMD to provide the three most plausible metabolite identity candidates according to spectral similarity and RI error. We confirmed the identity of 62 of these EI spectra (see Section 2 for details). Of the 62 identified EI spectra, only 44 cases had at least two candidates with both spectral and RI reference data. All the candidates in those cases (a total of 106 metabolites) were removed from GMD and kept as test set, and the remaining 1053 metabolites in GMD were employed to train the SVM linear model.

Figure 5b,c shows the experimental vs. reference RI and the experimental vs. predicted RI for the cases where the identified EI spectra had at least two candidates with both spectral and RI reference data. We compared the experimental with the predicted RI error to assess the predicted RI accuracy. The experimental relative RI error was computed as the relative error between the empirically calculated RI by eRah and the reference RI from GMD (observed vs expected RI). The predicted RI error was computed as the relative error between the empirically calculated RI by eRah and the predicted RI by the SVM prediction model. Mean and median experimental relative RI errors were 0.52% and 0.49%, respectively; whereas the mean and median predicted relative RI errors were 2.55% and 2%, respectively. Median and mean experimental absolute RI errors were 7.35 and 6.64 RI units, whereas the mean and median predicted absolute RI errors were 35.6 and 25.6 RI units, respectively.

Next, we focused on the model’s ability to rank the true candidate from the most to the least plausible identity according to the RI error. Spectral similarity, the most widely used method for ranking putative candidates in MS [38,39], ranked the true metabolite identity the first (most similar spectra) among the three candidates in 37 out of 44 cases; whereas in the rest of the cases (7 out of 44), the true identity was the second candidate (second most similar spectra). Instead, the experimental and predicted RI error ranked the true metabolite identity the first (lowest error) among the three candidates in 41 and 36 cases, respectively (Figure 5). This corresponds to an accuracy of 84% for the spectral similarity, 93% for the experimental RI error and 82% for the predicted RI error.

Finally, we retrieved the statistically significant differences between UC and the control group. Figure 5d shows the heat-map and the samples hierarchical clustering built with the 62 identified metabolites. To account for sex metabolism differences, we compared the relative abundances of the identified metabolites in UC males with control males and UC females with control females. Hydroxylamine was statistically significant dysregulated in UC males (*p*-value < 0.05, *t*-test) whereas none of the identified metabolites were statistically significant dysregulated in UC females. Of note, we removed all metabolites showing a coefficient of variation greater than 20% in QC samples, following established untargeted metabolomics data analysis guidelines [40]. Despite the pilot-study nature of our untargeted analysis (consisting of small sample size), these results suggest a rather distinct sex-related metabolic profile in UC.

## 4. Discussion

We compared different combinations of ML methods and FP classes generated by different calculation software. Specifically, we compared SVM with DNN, CNN and RF prediction models. The best results were obtained by an SVM model using a linear kernel and trained with ECFP-class FPs generated with Dragon (now discontinued but replaced by alvaDesc). SVM-based models showed similar performance whereas RF and CNN methods yielded lower accurate predictions compared to the other methods. Previous studies reported that RF outperformed ANN (forerunner algorithm of DNN) when trained only with specific compound classes (polycyclic aromatic hydrocarbon [30] or sulfur organic [31] compounds), but the structural heterogeneity of metabolites in our model could have affected the RF ability to accurately predict RI values. CNN algorithm benefits from large datasets, suggesting that the low number of metabolites employed to train the model might not be enough to achieve the same performance as the other models. Interestingly, our results showed similar prediction accuracy of CNN and RF, despite that CNN is one of the most computationally expensive models and with a complex architecture, whereas RF architecture is one of the simplest. Also, the FP type and calculation software used as input for the ML model yielded different prediction performances. The use of ECPF generated with Dragon clearly outperformed the rest of the FP classes. Both Dragon- and RDKit-generated ECFP-class FPs presented different accuracy results, demonstrating that model performance is strongly affected not only by the FP class but also by the FP calculation software and consequently by the underlying calculation algorithm.

Previous studies have reported that similar molecular structures have a similar RT. We focused on assessing whether these observations were echoed in GC–MS RI prediction. First, the same metabolites with different TMS groups (e.g., leucine 1TMS and leucine 2TMS) share a common molecular structure. Based on the assumption that molecules differing only by the number of TMS groups should share a similar RI difference, we used a simple linear regression among RI values of metabolites with 1 and 2, and 2 and 3 TMS groups (Figure 2a,b). Surprisingly, this procedure was as accurate as the SVM model trained with certain FPs, although its use is limited to metabolites for which the RI of at least one TMS derivative is know. The structural similarity also has an influence on the prediction performance when using ML-based models, and is mainly modulated by the training set. Figure 3b,c show that the prediction accuracy increases when there is at least one similar metabolite in the training set—the more similar the structure is, the lower the prediction error is. This demonstrates that the structural similarity between the training and test set modulates the prediction performance. Based on these observations, it is intuitive to think that we can estimate the expected prediction error interval based on the structural similarity of a metabolite in real cases based on its similarity with metabolites in the training set. Thus, we determined the probability of prediction error for a given metabolite being lower than a specific threshold depending on the metabolite’s structural similarity to the metabolites in the training set. We observed that the probability of obtaining a prediction error lower than 1% is 48% but only when the structural similarity is between 90 to 100%. Using less restrictive premises, e.g., structural similarity of at least 70% and a probability of 80%, the expected prediction error is lower than 5.7%. This probability estimation approach can be used to estimate the prediction error range but its limitations include the model’s inherent accuracy, and the fact that it takes into account the observed error of only the most similar metabolite. A more advanced statistical approach considering the observed errors of multiple similar molecules could provide more accurate error estimations.

We demonstrated the application of our prediction model in two GC–MS-based untargeted metabolomics cases: simulating a case where there are several putative candidates with similar mass (CI) or similar spectra (EI) (Figure 4), and in a metabolite identification case in plasma samples (Figure 5). Our results showed the potential application of predicted RI in metabolomics when multiple candidates match to a monoisotopic mass or spectrum. Even with more than 4 putative candidates with the same molecular formula, predicted RI values enabled ranking the correct metabolite identity among the top three candidates (those with the lowest predicted-experimental RI error) in up to 50% of the cases. Conversely, the prediction model showed a modest-to-poor filtering capacity as demonstrated by the ROC curve analysis, with AUC values of 0.53 and 0.56 for the CI and EI cases, respectively. Also, using a 3% predicted-experimental RI error filtering threshold (as determined by ROC) we reduced the number of putative candidates to nearly half, but at the cost of retaining a large number of false identities—nearly as many as retained true identities—and filtering out a large number of true identities. Overall, These observations are in alignment with what has been previously observed also in large-scale liquid chromatography RT prediction models [15].

The application in real samples allowed us to compare the differences between experimental to reference and experimental to predicted RI errors. Previous studies have reported an absolute RI difference of 7.6 RI units when comparing reference with experimental data [41]; and a relative RI error of 0.5 to 1% has been proposed as an identification threshold [42]. Our prediction model yielded median absolute and relative errors of 37.1 RI units and 2%, which suggest that considering only predicted RI values can not be used for unambiguous identification but can provide insights into the true structure among putative candidates with similar spectra by ranking from the most to the least plausible true identity. This is of special importance to identify known metabolite structures that lack reference spectra and RI. In these cases, putative metabolite identities could be narrowed to the most likely by combining CI (which provides the monoisotopic mass) and predicted RI. The model also showed a capacity at ranking the true metabolite identity among other candidates similar to the use of reference data or the use of MS spectral data. Interestingly, the model showed a greater capacity at ranking the true metabolite identity among other candidates in plasma samples compared to the simulated case. This is because our simulated case takes into account all possible cases, thus including the worst-case scenario where we observe a large number of metabolites with isomeric counterparts (Figure 3a). Isomeric molecules share a similar RI and are more difficult to rank according to their RI. Metabolites identified in the plasma samples largely consist of metabolites that do not have isomeric counterparts or these counterparts are not included in the library, thus facilitating the ranking of true candidates among a more structurally heterogeneous pool of candidates. This also demonstrates the importance of using simulated cases to assess the generalization power of the hypotheses and observed results in real cases.

## 5. Conclusions

We evaluated the accuracy of different ML models at predicting the retention time of trimethylsilyl derivatives of metabolites in GC–MS, through the use and prediction of RI (relative retention time). We compared five popular ML methods trained with different molecular FPs as input data. We conclude that the model performance is affected by the molecular FP class employed to represent the compound structure and also by the software employed to generate these FPs. We proposed a model based on an SVM with a linear kernel trained with ECFP-class FP generated with the Dragon software. New alternatives like those based on graph neural networks and that bypass FP calculation could potentially yield better prediction accuracy [43]. Independently, the model is ultimately affected by the structural similarity of metabolites used to train the model to those in a real case, as this is an inherent limitation of machine learning.

Our study, that challenged predicted RI when using CI and EI ionization sources in simulated and real cases, demonstrated a good true identity ranking capability of our RI prediction model among all candidates, despite its modest-to-poor false identity filtering power, which is in alignment with what has been previously observed in large-scale RT prediction studies [15]. Also, ranking performance using predicted RI values in experimental samples was comparable to the performance obtained when using spectral similarity, which suggests that the combined use of both reference spectral data and predicted RI could enhance the overall accuracy in cases where only reference spectral data are available.

Collectively, our study provides a rationalized framework for machine learning-based retention time or RI prediction of TMS derivatives of metabolites in GC-MS, of special utility in cases where reference data or commercial standards are unavailable.

## Data Availability

The R scripts to reproduce the results are available at https://github.com/smdecripan/RIpred (accessed on 1 March 2022). GC–MS raw data (mzXML files) has been deposited in the Metabolights repository with accession number MTBLS2841.

## Supplementary Materials

The following supporting information can be downloaded at: https://www.mdpi.com/article/10.3390/biomedicines10040879/s1, S1 Metabolite Extraction Method; S2 GC-MS analysis; S3 Model parameters; S4 Fingerprint classes used [44]; S5 CE and EI simulation and validation; Figure S1: Receiver operating characteristic (ROC) curves; Table S1: Fingerprint (FP) classes notation, software and classification summary [45,46]; Table S2: Performance of the prediction models depending on ML model and FP class.
