# Supplementary material for: Machine Learning-Based Retention Time Prediction of Trimethylsilyl Derivatives of Metabolites
Source: Biomedicines. 2022 Apr 11;10(4):879. doi: 10.3390/biomedicines10040879 (PMC9024754; doi:10.3390/biomedicines10040879)
Supplement: Supplementary file 1 [file biomedicines-10-00879-s001.zip › biomedicines-1663567-supplementary.pdf]

Machine learning-based retention time prediction of trimethylsilyl derivatives of metabolites

Sara M. de Cripan [1,2,3], Adria Cereto-Massague [2], Pol Herrero [2], Andrei Barcaru [4], Nuria Canela [2] and Xavier Domingo-Almenara [1,2,3,*]

1 Computational Metabolomics for Systems Biology Lab, Omics Sciences Unit, Eurecat—Technology Centre of Catalonia, Barcelona, 08005, Catalonia, Spain; sara.martinez@eurecat.org

2 Centre for Omics Sciences (COS), Eurecat—Technology Centre of Catalonia & Rovira i Virgili University Joint Unit, Unique Scientific and Technical Infrastructures (ICTS), Reus, 43204, Catalonia, Spain; adria.cereto@ce.eurecat.org (A.C.-M.); pol.herrero@eurecat.org (P.H.); nuria.canela@eurecat.org (N.C.)

3 Department of Electrical, Electronic and Control Engineering (DEEEA), Universitat Rovira i Virgili, Tarragona, 43007, Catalonia, Spain

4 Independent Researcher, Amsterdam, 1012 WX, The Netherlands; andrei_barcaru@yahoo.com

* Correspondence: xavier.domingoa@eurecat.org

Supporting Information Contents:

S1 Metabolite Extraction Method 1

S2 GC-MS analysis 1

S3 Model parameters 1

S4 Fingerprint classes used 2

S5 CI and EI simulation and validation 2

S6 Supplementary tables 3

S7 Supplementary figures 4

S1 Metabolite Extraction Method

Serum aliquots (25 µL) were thawed at 4°C. Samples were briefly vortex-mixed and each aliquot was supplemented with 20 µL of 1 µg/µL succinic- d4 acid (internal standard). Proteins were then precipitated by the addition of 475 µL of cold methanol/water (8:1 vol/vol) followed by 3 min of ultrasonication and 10 s of vortex-mixing. Aliquots were subsequently maintained on ice for 10 min. After centrifugation for 10 min (19000 g, 4°C), 100 µL of supernatant were transferred to a GC autosampler vial and lyophilized. We incubated the lyophilized serum residues with 50 µL of methoxyamine in pyridine (40 µg/µL) for 30 min at 60°C. To increase the volatility of the compounds, we silylated the samples using 30 µL of N-methyl-N-trimethylsilyltrifluor- oacetamide with 1% trimethylchlorosilane (Thermo Fisher Scientific) for 30 min at 60°C.

S2 GC-MS analysis

The GC–TOF system consisted on a 7890 gas chromatograph from Agilent equipped with a HP-5 column (30 mm x 0.25 mm i.d., 0.25 µ), coupled to a Pegasus 4D mass spectrometer from LECO Corporation (St. Joseph, MI, USA). 1 µL of the derivatized plasma extracts were injected in a split/splitless inlet at 250 ±C in split mode, at 1:5 split rate and a constant He flow of 1mL/min. Initial oven temperature was set at 70 ±C, held for 1 min, raised at 10 mL/min to 325 ±C and held at the final temperature for 10 min. The MS spectrometer operated in electron impact mode at 70 eV. Spectral data was acquired after 4 min of solvent delay, in full scan mode from m/z 35 to 700 at an acquisition rate of 10 spectra/s. Transfer line and MS source were both at 250 ±C.

S3 Model parameters

We performed hyperparameter tuning for each of the models, the parameters described below correspond to those that demonstrated higher accuracy with Dragon 7 generated fingerprints as input data. Although they are not included, for SVM model with linear kernel we also performed hyperparameter tuning varying the initial data for each fingerprint class but there was no significant improvement in the precision of the model, being those generated with Dragon7 those that gave the best results over the rest.

We used a DNN model with two fully connected layers with ReLU activation functions, followed by a global average pooling layer with a single unit. First layer's input shape was defined by the total number of bits encoded by each class of fingerprint and the model was initiated with 200 units, the second layer contained 64 units. The DNN model was compiled using RMSprop optimizer.

For CNN model we alternated three 1d convolutional layers, with ReLu activation and kernel size set in 6, with average pooling layers, with a pool size of 4. First convolutional layer was composed of 600 filters and as well as for the DNN model, input shape was determined by the total number of bits defined by each class of finger-print. Second and third convolutional layers contained 320 and 164 filters respectively. Finally, two dense layers with ReLU activation with 64 and 1 units respectively concluded the model architecture. CNN model was compiled with RMSprop optimizer and it required 130 epochs and a batch size of 1 for each gradient to complete the training task.

RF model was composed of 600 trees and the importance of predictors was not assessed considering all the variables.

SVM models were built with two different kernel functions all of them were used as epsilon regression machine: SVM-Lin (kernel = "linear", epsilon = 0.1, cost = 100) and SVM-Poly (kernel = "polynomial", cost = 1, degree = 6, coef0 = 6, epsilon = 0.1).

For SVM with linear kernel we evaluated how molecular structure similarity across molecules allocated in training and test set impact on prediction accuracy of RI values. For each molecule contained in the test sets we selected the molecule in the training set with maximum Tanimoto similarity coefficient. We evaluated RI prediction error by grouping test metabolites according to the Tanimoto value. Mann-Whitney-Wilcoxon test was applied with R-base functions to each group.

S4 Fingerprint classes used

FP classes employed can be classified in circular, substructure keys-based and topological or path based depending on the method employed to transform molecular representation into a bit string[1]. Extended-Connectivity Fingerprints (ECFPs) are popular circular-class FP that represent circular atom neighbourhoods up to a specified bound radius from the central atom. We employed RDKit to generate ECFP2 and ECFP4 with 2 and 4 diameter respectively and ECFPs generated with commercial software Dragon 7. Substructure keys-based fingerprints set the bits according to the absence/presence in the compound of specific substructures given in a list of structural keys. PubChem FP (Pb) include 881 structural keys whereas MACCS FP are 166 length binary vectors[2], both FP classes generated with ChemFP and OpenBabel as back-end. FP2 is a path-based FP generated with OpenBabel (referred as OBFP2), a compound structure is analysed to identify linear fragments with 1-7 atoms length, ignoring C, N and O atoms, when the atoms form a ring the fragment is terminated and only canonical fragments are retained. Layered fingerprints are a topological based experimental FP implemented in RDKit, molecular structure is depicted by layers that measure atom and bond types contributions. For all bit-string FPs, except MACCS that preserved 166 bits length, a size of 1024 bits was used. See Table S1 for more information about FP classes and notations.

S5 CI and EI simulation and validation

We evaluated the application of predicted RI in the two widely used MS ionization methods in GC, chemical ionization (CI) and electron ionization (EI). In CI multiple candidates can be matched via accurate mass searching as well as in an EI case through metabolite's spectrum. We simulated the CI experimental scenario, randomly selecting 70 compounds that were considered the experimental compounds to be identified, for each one we selected those compounds with the same m/z within 10 ppm error that were considered the other putative candidates. In that sense, we evaluated the SVM-Lin model performance at ranking putative identities based on their predicted-actual RI error with a special focus on the capability to rank the correct identity among the top 3 first candidates with the minimum error. We used the resampling procedure for validation, similar to the described above (Figure 1), so this

yielded 20 validation sets with different sizes that span from 143 to 197 (median 179.5, which corresponds to a 15% of the sample size). A similar procedure was used for the EI case: 20 random molecules were used to initialize the validation set and for each of these molecules we searched for other molecules with a similar spectra (similarity of 80% or above using the cosine dot product). This procedure allowed to simulate the scenario in which a spectra can match multiple similar spectra in the database. For the EI case, this procedure yielded 20 validation sets with different sizes that span from 44 to 142 (median 94.5, which corresponds to a 8% of the data).

We evaluated the SVM-Lin model filtering capability over the first 3 candidates with minimum RI error in simulation conditions of CI and EI. We established a threshold value to determine the true identity of the candidates. We considered a metabolite classified as true positive if it presents an error below the established threshold and is the considered metabolite, by contrast, if it presents an error below the threshold but it does not correspond to the metabolite identity was considered as false positive. We classified as false negative metabolites that correspond to the true metabolite identity but the error is above the established threshold and as true negative those metabolites that were correctly discarded with an RI error above the threshold. To determine the best threshold, we used a receiver operating characteristic curve (ROC) calculated for the RI error range from 0 to 100% in intervals of 1% (Figure S1). For each error threshold we calculated the True Positive Rate (TPR) and False Positive Rate (FPR) to deduce the threshold value to find the balance between filtering the largest number of false positives without sacrificing true positives.

S6 Supplementary tables

Table S1: Fingerprint (FP) classes notation, software and classification summary.

Notation	Software	FP class	Classification	Reference
Dragon7	Dragon7	ECFP	Circular	[3]
ECFP2	RDKit	ECFP $\varnothing = 2$	Circular	[4]
ECFP4	RDKit	ECFP $\varnothing = 4$	Circular	[4]
Layered	RDKit	Layered	Topological	[4]
OBFP2	OpenBabel	FP2	Topological	[5]
MACCS	ChemFP (OpenBabel as back-end)	MACCS	Substructure keys-based	[5, 6, 7]
PB	ChemFP (OpenBabel as back-end)	PubChem	Substructure keys-based	[5, 6, 7]

Table S2: Performance of the prediction models depending on ML model and FP class. MAE, Mean Average Error; MAPE, Mean Average Percentage Error; MdAE, Median Average Error; MdAPE, Median Average Percentage Error.

Model	FP class	MAE	MAPE (%)	MdAE	MdAPE (%)
DNN	Dragon7	66.07	3.46	42.08	2.18
CNN	Dragon7	101.93	5.27	74.92	3.90
RF	Dragon7	105.20	5.50	70.18	3.61
SVM-Poly	Dragon7	62.98	3.26	36.50	1.88
SVM-Lin	Dragon7	62.53	3.26	37.13	1.95
SVM-Lin	ECFP2	195.54	10.07	115.95	6.21
SVM-Lin	ECFP4	218.28	11.56	141.67	7.33
SVM-Lin	layered	176.30	8.95	96.97	5.11
SVM-Lin	MACCS	237.18	12.06	162.53	8.81
SVM-Lin	OBFP2	207.84	10.49	115.91	6.04
SVM-Lin	PB	262.16	14.18	148.71	7.91

S7 Supplementary figures

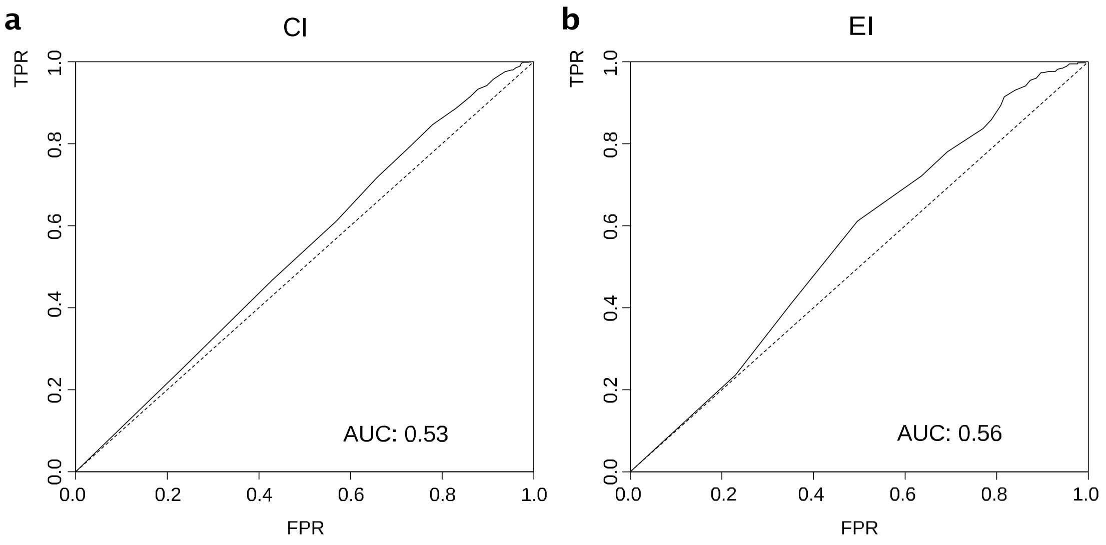

Figure S1. Receiver operating characteristic (ROC) curves constructed calculating the true positive rate (TPR) and false positive rate (FPR) for RI relative error thresholds from 0 to 100% in intervals of 1% for **a** CI case and **b** EI case. AUC refers to Area Under the Curve.

Supplementary references

[1] Cereto-Massagué, A.; Ojeda, M.J.; Valls, C.; Mulero, M.; Garcia-Vallvé, S.; Pujadas, G. Molecular fingerprint similarity search in virtual screening. Methods 2015, 71, 58–63. doi:10.1016/j.ymeth.2014.08.005.

[2] Riniker, S.; A Landrum, G. Open-source platform to benchmark fingerprints for ligand-based virtual screening. J. Chemin- 2013, 5, 26–26, https://doi.org/10.1186/1758-2946-5-26.

[3] K. srl., Dragon (Software for Molecular Descriptor Calculation) version7.0 (https://chm.kode-solutions.net/,accessed on 1 March 2022).

[4] Landrum, G. RDKit (Open-Source Cheminformatics Software) 2021. (https://www.rdkit.org/).

[5] O'Boyle, N.M.; Banck, M.; James, C.A.; Morley, C.; Vandermeersch, T.; Hutchison, G.R. Open Babel: An open chemical toolbox. J. Cheminformatics 2011, 3, 33. doi:10.1186/1758-2946-3-33.

[6] Dalke, A. ChemFP (Software for Fingerprint Generation and High-performance Similarity Search,https://chemfp.com/, accessed on 1 March 2022).

[7] Dalke, A. The chemfp project. J. Cheminformatics 2019, 11, 76. doi:10.1186/s13321-019-0398-8.
